# Targeted deletion of NFAT-Interacting-Protein-(NIP) 45 resolves experimental asthma by inhibiting Innate Lymphoid Cells group 2 (ILC2)

**DOI:** 10.1038/s41598-019-51690-z

**Published:** 2019-10-30

**Authors:** Sonja Koch, Lisa Knipfer, Julia Kölle, Hooman Mirzakhani, Anna Graser, Theodor Zimmermann, Alexander Kiefer, Volker O. Melichar, Wolfgang Rascher, Nikolaos G. Papadopoulos, Ralf J. Rieker, Benjamin A. Raby, Scott T. Weiss, Stefan Wirtz, Susetta Finotto

**Affiliations:** 1 Department of Molecular Pneumology, Friedrich-Alexander University Erlangen-Nürnberg, Erlangen, Germany; 2 Department of Medicine 1 - Gastroenterology, Pneumology and Endocrinology, Friedrich-Alexander-University Erlangen-Nürnberg, Erlangen, Germany; 3 Channing Division of Network Medicine, Brigham and Women’s Hospital, Harvard Medical School, Boston, MA USA; 4 Department of Pediatrics and Adolescent Medicine, Universitätsklinikum Erlangen, Erlangen, Germany; 5 Allergy and Clinical Immunology Unit, 2nd Pediatric Clinic, National and Kapodistrian University of Athens, 11527 Athens, Greece; 6 Division of Infection, Immunity & Respiratory Medicine, University of Manchester, Manchester, UK; 7 Institute of Pathology, Friedrich-Alexander-Universität Erlangen-Nürnberg (FAU), Erlangen, Germany

**Keywords:** Respiratory signs and symptoms, Chronic inflammation, Molecular medicine

## Abstract

Here we investigated the role of NFAT-interacting protein (NIP)-45, an Interleukin (IL)-4 inducing Transcription Factor, and its impact on the differentiation of Group 2 Innate -Lymphoid -Cells (ILC2s) in the pathogenesis of asthma. *NIP45*, a transcription factor regulating NFATc1 activity, mRNA was found to be induced in the Peripheral Blood mononuclear cells (PMBCs) of asthmatic pre-school children with allergies and in the peripheral blood CD4^+^ T cells from adult asthmatic patients. In PBMCs of asthmatic and control children, *NIP45* mRNA directly correlated with NFATc1 but not with T-bet. Targeted deletion of NIP45 in mice resulted in a protective phenotype in experimental asthma with reduced airway mucus production, airway hyperresponsiveness and eosinophils. This phenotype was reversed by intranasal delivery of recombinant r-IL-33. Consistently, ILC2s and not GATA3^+^ CD4^+^ T-cells were decreased in the lungs of asthmatic NIP45^−/−^ mice. Reduced cell number spleen ILC2s could be differentiated from NIP45^−/−^ as compared to wild-type mice after *in vivo* injection of a microcircle-DNA vector expressing IL-25 and decreased cytokines and ILC2 markers in ILC2 differentiated from the bone marrow of NIP45^−/−^ mice. NIP45 thus emerges as a new therapeutic target for the resolution of the airway pathology, down-regulation of ILC2s and mucus production in asthma.

## Introduction

Allergic asthma is a disease associated with reversible airway inflammation, airway hyperresponsiveness (AHR), mucus production and airway remodeling. All these features have been linked to an aberrant differentiation of T helper (h)2 cells that produce the cytokines IL-4, IL-5 and IL-13^1^. Current treatments for allergic asthma are anti-inflammatory medications enclosing steroids^2^. Nuclear factor of activated T-cells(NFAT)-interacting protein (NIP)45 is a Th2 associated transcription factor known to potentiate Nuclear factor of activated T-cells (NFATc)2-driven interleukin (IL)-4 expression^3^. After T cell receptor antigen challenge, NFAT-interacting protein (NIP)-45 is methylated at its amino-terminal region by the protein arginine methyltransferase (PRMT1). The arginine methylation domain of NIP45 supports the interaction with nuclear factor of activated T cells (NFAT) and recruits PRMT1 to the NFAT transcription-activating complex, thereby enhancing the production of IL-4^4^. Moreover, NIP45 deficient mice have been shown to be deficient in IL-4 and IFN-γ production, indicating that NIP45 controls both Th1 and Th2 cytokine production^3–5^.

NFAT is a family of transcription factors that are known to be activated by calcium influx and consist of five family members (NFATc1, NFATc2, NFATc3, NFATc4 and NFAT5)^6^. In activated T cells, NFATc1 was first identified to control the IL-2 promoter^7^. Individual family members play different roles in T cell development and activation. Additionally, they also have effects on the regulation of numerous other immune cells^8–11^.

The different NFAT family members regulate Th1, Th2 and Th17 cells^12^. It has been previously shown that in the absence of NFATc2, the differentiation of Th1 cells is diminished, while the development of Th2 cells and IL-4 production is up-regulated, especially in a model of allergic asthma^9,13^. The loss of both NFATc2 and NFATc3 leads to increased expression of Th2 cytokines as well as to high IgG1 and IgE levels^14–17^. In contrast, we and others have demonstrated that the absence of NFATc1 in T cells, IL-4 and other Th2 cytokines as well as the levels of IgE and IgG1 production are reduced^16,18,19^. Thus the phenotype of NFAT deficient mice does not reflect differences in DNA-binding or transcriptional activity at the IL-4 promoter, but most likely indicates interactions with other regulating transcription factors or other indirect effects.

Therefore, we reasoned that NFAT regulating transcription factors like NIP45 may be involved in the regulation of NFAT and thus in the development of allergic asthma^1^.

Innate lymphoid cells group 2 (ILC2) do not express lineage markers but share effector functions with Th2 cells. They are a rare cell population that comprises a distinct lineage of IL, found to be increased after allergen treatment. They are able to develop type-2 immune responses^20–23^. ILC2s differentiate in the bone marrow from a common lymphoid precursor (CLP)^24,25^. It has been demonstrated that they express ST2, which is a receptor subunit of IL-33, IL-17RB, a receptor subunit of IL-25, and TSLPR, which is a component of the receptor for thymic stromal lymphopoietin^21^. Therefore, ILC2s are cells that respond to alarmin-like cytokines such as IL-33, IL-25 and thymic stromal lymphopoietin (TSLP) which are released by activated or damaged epithelial cells. Epithelial cells are the first barrier of defense against allergens and play a crucial role in the development of allergic asthma^26,27^. Furthermore, ILC2s express the common γ-chain CD132, as well as CD25 and CD127. For the development and function of ILC2s the transcription factors GATA3 and RORα play essential roles. In the lungs, ILC2s have a critical role in inducing an innate type 2 immune response caused by inhaled allergens^28,29^. They are the primary source of the cytokines IL-5 and IL-13. Therefore they represent an important cell type in diseases mediated by type 2 immune responses, such as asthma and allergy^22,28,30–32^.

The aim of this study was to better understand the function of NIP45 in allergic asthma and its possible impact on the biological functions of ILC2s. We observed that NIP45^−/−^ mice show decreased pathological characteristics of asthma, like inflammation, AHR and mucus production, in two experimental asthma models after OVA and HDM challenge, respectively. Furthermore, we detected a defect in ILC2 survival and proliferation in the absence of NIP45 in allergic asthma and spleen derived ILC2s, indicating a crucial role for NIP45 in mediating asthma via ILC2.

## Results

### Increased expression of *Nip45* mRNA in asthmatic subjects positively correlated with NFATc1 levels

**Figure 1:**
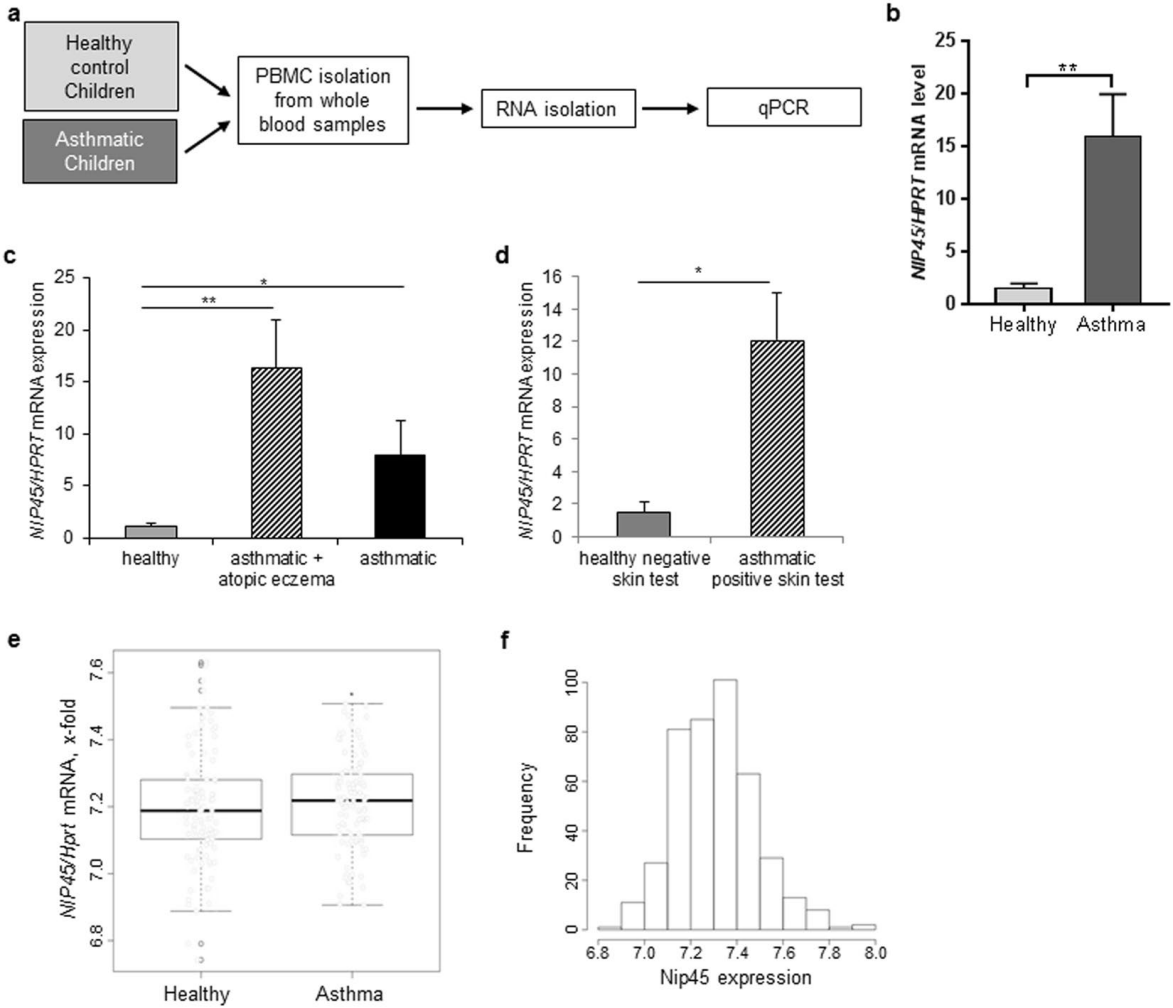
Increased expression of Nip45 in children with asthma. (**a**) Experimental design of PBMCs RNA isolation for qPCR from healthy and asthmatic children. (**b**–**d**) Relative mRNA expression for NIP45. n = 12–17 children per group. (**e**,**f**) Differential NIP45 mRNA expression between asthmatics and healthy controls in Asthma BRIDGE study (p < 0.001 obtained from Wilcoxon rank sum test with continuity correction; N.healthy = 122, N.asthmathics = 300). Distribution of NIP45 mRNA expression among 422 subjects.

**Figure 2:**
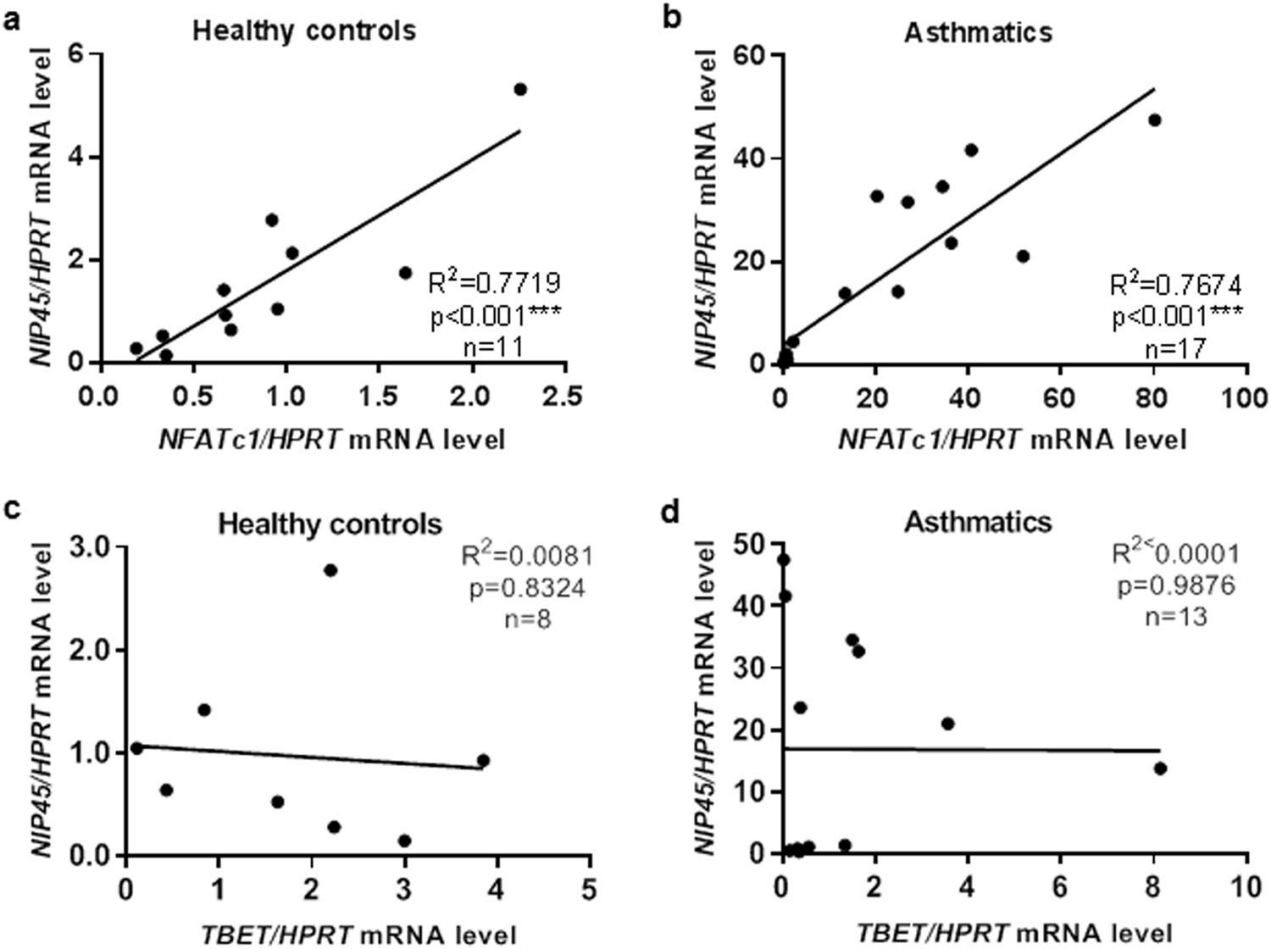
*NIP45* mRNA directly correlated with NFATc1 mRNA but not with *T-bet* mRNA in PBMCs of control and asthmatic pre-school children. (**a**,**b**) Linear regression analysis of qPCR analysis for *NFATc1* and *NIP45* mRNA corrected by HPRT mRNA expression of the cohorts of Predicta children described in panels a and b. Healthy controls n = 11, asthma n = 17. In the same children a correlation between NIP45 and T-bet mRNa was performed (**c**,**d**).

In the context of the European Study PreDicta, we examined two cohorts of healthy and asthmatic pre-school children at the age of 4–6 years (Table S1). The expression of *NIP45* mRNA in unstimulated PBMCs from these two cohorts of pre-school children was then analyzed. Here we observed that children with asthma expressed significantly higher *NIP45* mRNA levels than healthy individuals (Fig. 1a,b and Table S1). In table S1 also the medications taken by the children with asthma are reported. No relation was observed between patients taking steroids and those treated with non-steroid medication and NIP-45 expression. We next analyzed the expression of NIP45 in PBMCs from these asthmatic children with additional self-reported atopic eczema. NIP45 was found significantly induced in asthmatic pre-school children with self-reported atopic eczema and positive skin test (Fig. 1c,d, respectively), similarly to what we recently reported for NFATc1 expression in these cohorts of children. To confirm these findings, in a second cohort of subjects from the Asthma Bio-Repository for Integrative Genomic Exploration (ABRIDGE, N_asmathics_ = 300, N_healthy_ = 122), we investigated the mRNA expression of *NIP45* in peripheral blood CD4+ T cells. After adjustment for age, race, gender and batch effect, the average *NIP45* mRNA expression was moderately higher among asthmatics than non-asthmatic controls (p for moderated t-statistics = 0.036, fold change = 1.04, Fig. 1e,f). These findings were consistent with the idea that NIP45 might have a role in asthma. Moreover, the increase in NIP45 seen in PBMCs of asthmatic children shows a ~5 fold expression difference, whereas in sorted CD4+ T cells this is only 1.04-fold. These findings are consistent with a role of NIP45 expression in Th2 cells but also in other cell type present in the PBMCs of asthmatic children. Furthermore, we next asked about a correlation between the recently described increase of NFATc1 in the blood of children with asthma and NIP45. Therefore, we next analyzed the correlation between NIP45 and NFATc1 mRNA expression in the blood cells of these children and found a highly significant direct correlation between the expression levels of these two transcription factors both in healthy controls and in asthmatic children (Fig. 2a,b, respectively).

This direct correlation was not observed when *NIP45* mRNA was correlated with T-bet, (Fig. 2c,d), another protein present on the promoter of IFN-gamma closely associated with *NFATc1*^23^, mRNA extracted from the PBMCs of asthmatic and control children, indicating a specific role of Nip45 in association with NFATc1 in allergic asthma (Fig. 2c,d).

### NIP45 deficiency is associated with a reduced asthmatic phenotype

**Figure 3:**
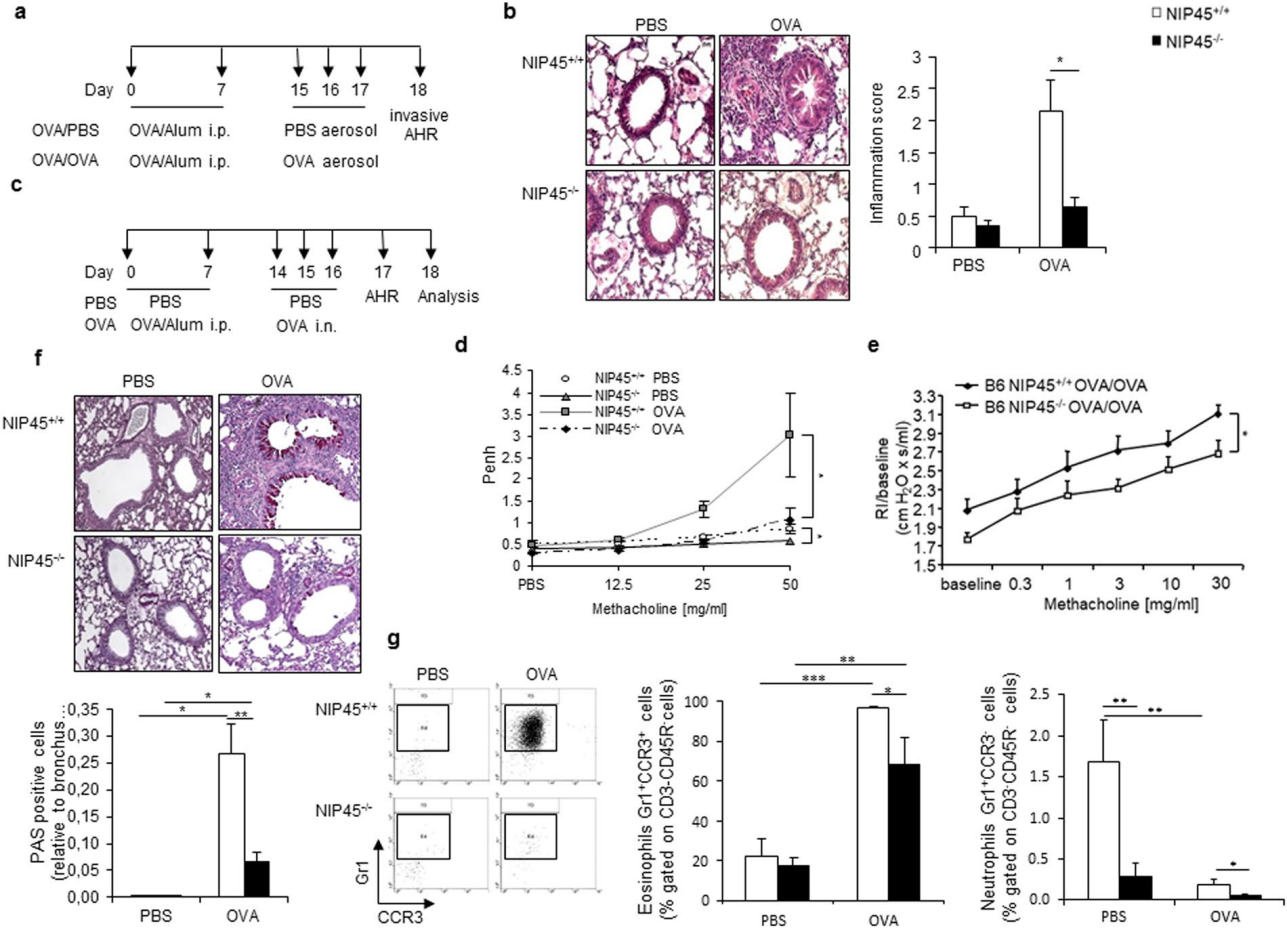
Inhibition of the asthmatic phenotype in Nip45^−/−^ mice in a murine model of allergic asthma. (**a**) Experimental design of the murine model of allergic asthma for the analysis of the invasive plethysmography and lung inflammation. (**b**) Histological sections of the lungs of non-asthmatic and asthmatic wild-type and Nip45^−/−^ stained with H&E and corresponding pathological score of the inflammation. (**c**) Experimental design of the murine model of allergic asthma. (**d**) Airway resistance was analyzed with a non-invasive plethysmography as Penh and invasive method (**e**). (**f**) Periodic acid-Shiff (PAS) staining of lung sections of untreated and asthmatic Nip45^−/−^ and wild-type control mice to show mucus producing cells in purple. (**g**) The percentage of eosinophils (Gr1^+^CCR3^+^) and neutrophils (Gr1^+^CCR3^-^) in BALF of allergen sensitized and challenged Nip45^−/−^ and wild-type control mice were measured by flow cytometry. One representative experiment is shown with three to four mice per group. Students t-test was used to evaluate statistical significances. *P ≤ 0.05, **P ≤ 0.01, ***P ≤ 0.001. Data are expressed as mean ± s.e.m.

To explore the functional role of NIP45 in asthma, NIP45^−/−^ mice and wild-type littermates were analyzed in an experimental model of allergic asthma induced by ovalbumin (OVA) sensitization alone and OVA sensitization and challenge (Fig. 3a).

Lung histological sections were evaluated for peri-bronchial and perivascular inflammation as previously described^33^. Increased numbers of inflammatory cells were detected in the bronchi and peribronchial vessels of OVA challenged wild-type but not in NIP45^−/−^ mice (Fig. 3b). We also analyzed mice in a different genetic background (Balb/c) in naïve and OVA sensitized and challenged mice and confirmed the reduction of OVA-dependent AHR in the absence of NIP45 (Fig. 3c–e). In fact, OVA sensitized and OVA sensitized and challenges NIP45^−/−^ mice revealed a significantly lower AHR as compared to the wild-type control asthmatic groups as measured both by PenH and airway resistance (Fig. 3d,e, respectively). We further assessed mucus production in the lungs of wild-type and NIP45^−/−^ mice by PAS staining (Fig. 3f). OVA treatment induced mucus production in both, wild-type and NIP45^−/−^ mice, but NIP45^−/−^ mice showed significantly decreased number of PAS^+^ cells in this setting. Furthermore, we asked which kind of inflammatory cells where regulated by NIP45 in asthma. Eosinophils, which are significantly upregulated in the Bronchoalveolar lavage fluid (BALF) in asthma in wild type mice, where found to be significantly downregulated in the absence of NIP45 in asthma (Fig. 3g, left and middle panel). Residual Eosinophils remained in the airways in the absence of NIP45. The role of eosinophils in asthma has been revised to enclose resident eosinophils which play a resolving role in allergic asthma^34^. Further studies might address the possibility that NIP45 deficiency is associated with resident eosinophils. In addition, also neutrophils, a less represented inflammatory cell type in the airways of asthmatic mice, was also found inhibited in the BALF in the absence of NIP45 in this model of asthma (Fig. 3g, left and right panel). Taken together, these results suggested that NIP45 plays a pathogenic role in allergic asthma by regulating airway inflammation, mucus production and airway hyperresponsiveness.

**Figure 4:**
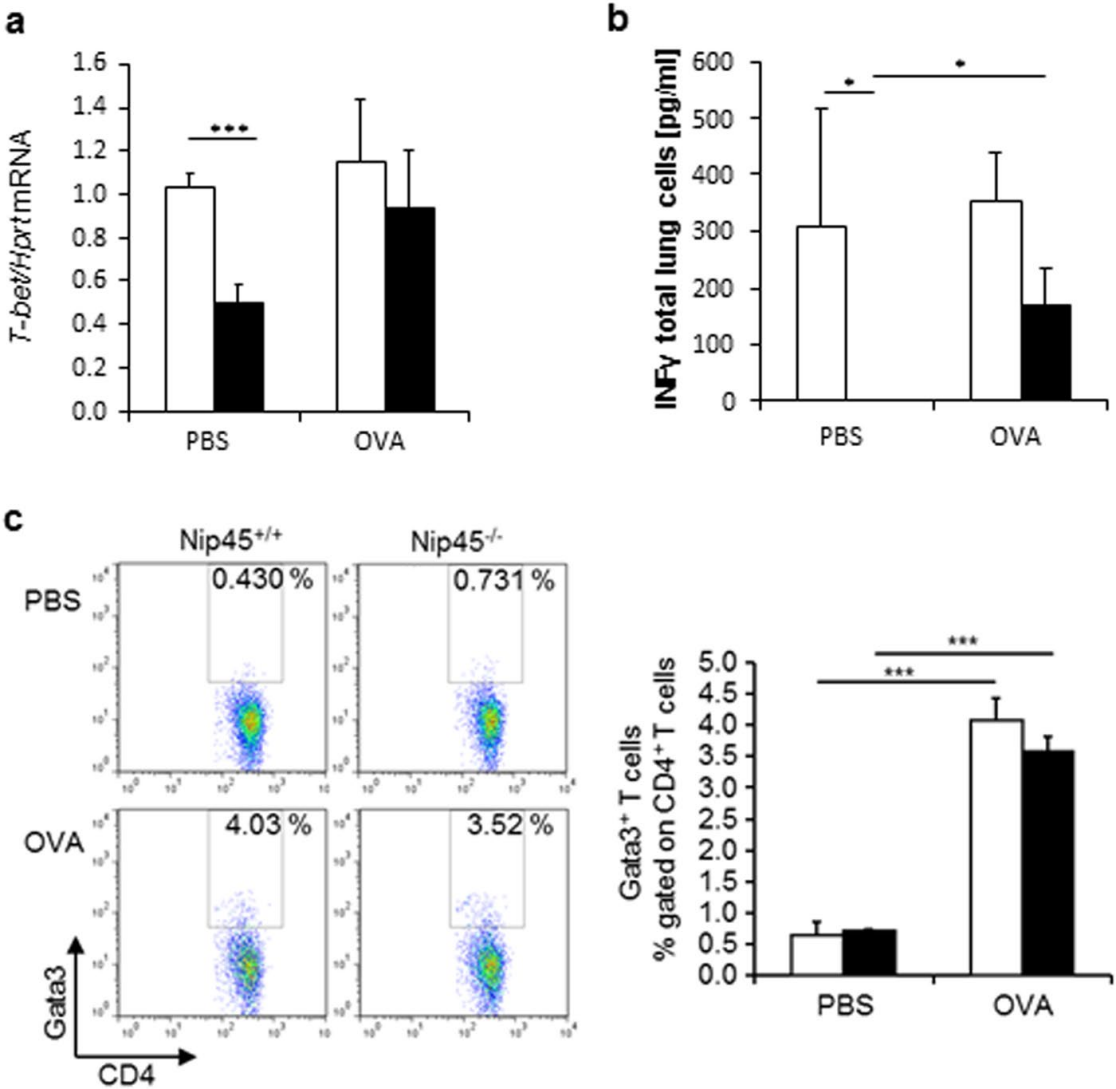
Decreased T-bet in the lung in the absence of NIP45. (**a**) *T-bet* mRNA was measured in the lung of wild type and NIP45^−/−^ mice. (**b**) INF-gamma in total lung cell supernatants of wild type and NIP45 deficient mice. (**c**) Percentage of CD4+ GATA3+ T cells in the lungs of Nip45^−/−^ and wild-type control mice analysed via flow cytometry. One representative of three independent experiments is shown with five to six mice per group. In this figure a Students t-test was used to evaluate statistical significances. *P ≤ 0.05, **P ≤ 0.01, ***P ≤ 0.001. Data are expressed as mean ± s.e.m.

We next analyzed *T-bet* mRNA in the lung of naïve and asthmatic wild type and NIP45^−/−^ mice. Here we found a downregulation of T-bet in the lung of naïve mice in the absence of NIP45(Fig. 4a). These results are consistent with a role of NIP45 on NFATc1 activitation on T-bet promoter^23^. In asthma, this effect was abolished probably because other transcription factors might replace NFATc1 on T-bet promoter. Consistent with a reported role of NIP45 on Th1^3–5^, targeted deletion of NIP45 resulted in absence of IFN-gamma in the airways (Fig. 4b). Consistent with T-bet, also IFN-gamma was reduced in naïve NIP45 deficient mice (Fig. 4b).

We next reasoned that, GATA-3 is a Th2 transcription factor which has been demonstrated to orchestrate airway inflammation, mucus production and airway hyperresponsiveness^35^. We then asked if lung CD4^+^ T cells expressing GATA-3 were regulated by NIP45 but although we detected a significant induction of GATA3^+^ CD4^+^ T cells in the OVA sensitized and challenged mice as compared to PBS mice, no difference was observed between wild type and NIP45^−/−^ mice (Fig. 4c).

### NIP45 deficiency leads to down regulation of Th2 cytokines

**Figure 5:**
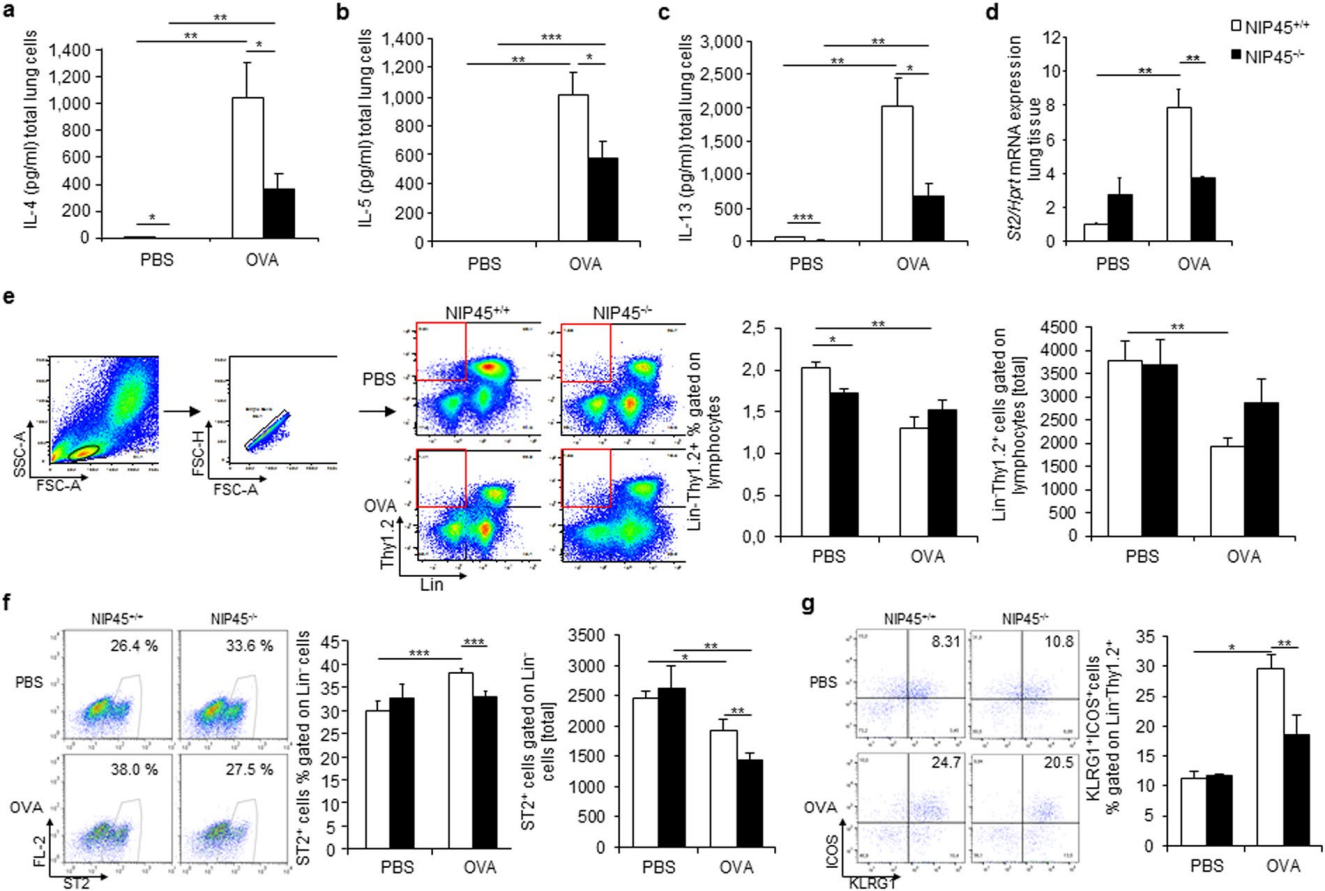
Decreased Th2 cytokine production and ILC2 numbers in the lungs of allergen sensitized and challenged Nip45^−/−^ mice. (**a**–**c**) Concentration of IL-4 (**a**), IL-5 (**b**) and IL-13 (**c**) in the supernatants of α-CD3/CD28 antibodies stimulated total lung cells isolated from Nip45^−/−^ and wild-type control mice after allergen sensitization and challenge measured via ELISA. One representative experiment is shown with three to four mice per group. (**d**) Expression of St2 mRNA in lung tissue of healthy and asthmatic wild-type and Nip45^−/−^ mice analyzed via qPCR. (**e**) Flow cytometry analysis of Lin-Thy1.2^+^ cells in total lung cells isolated from wild-type and Nip45^−/−^ mice after induction of asthma. (**f**,**g**) Analysis of Lin- ST2^+^ (**f**) and Lin- Thy1.2^+^ KLRG1^+^ ICOS^+^ ILC2s (**g**). n = 3–5 mice per group. Statistical significances in this figure were evaluated with a Students t test. * P ≤ 0.05, **P ≤ 0.01, ***P ≤ 0.001. Data are mean ± s.e.m.

Lymphocytes are a primary source of the type 2 cytokines IL-4, IL-5 and IL-13 which are upregulated in allergic asthma^36^. IL-4, a Th2 signature cytokine, was found significantly reduced in the supernatants of total lung cells isolated from naive and OVA treated NIP45^−/−^ mice compared to the wild-type control groups (Fig. 5a). Similarly, the production of IL-5 was also reduced in NIP45^−/−^ mice after sensitization and challenge with OVA compared to wild-type mice, while IL-5 protein was not detectable in total lung cells of untreated wild-type and NIP45^−/−^ mice (Fig. 5b). Analysis of IL-13 levels also revealed a significant increase in wild-type and NIP45^−/−^ mice after allergen sensitization and challenge. However, naive as well as asthmatic NIP45^−/−^ mice showed significantly lower levels of IL-13 compared to wild-type control mice (Fig. 5c). Collectively, these data indicate that NIP45 controls the inducible production of key type 2 signature cytokines in allergic asthma.

### NIP45^−/−^ asthmatic mice show a decreased percentage of ILC2s after allergen sensitization and challenge

ILC2s are innate like cells that increase after exposure to allergen and are known to express ST2, a member of the IL-1 receptor family^32^. They are innate lymphocytes known to be important producers of IL-5 and IL-13 and seem to play a role in the pathogenesis of allergic asthma^22,28,31^. We next investigated the expression level of *St2* mRNA in lung tissue from wild-type and NIP45^−/−^ mice before and after allergen sensitization and challenge (Fig. 5d). We observed that *St2* mRNA expression is significantly increased only in wild-type mice after induction of asthma. NIP45^−/−^ mice showed significantly reduced expression levels of *St2* mRNA in the OVA model compared to the wild-type littermates. We also investigated the number of Lin-Thy1.2^+^ cells in total lung cells. Thy1.2 (CD90.2) is a glycosylphosphatidylinositol (GPI)-anchored membrane glycoprotein involved in signal transduction and involved in co-stimulation of lymphocytes, all thymocytes, peripheral T cell proliferation and induction of hematopoietic stem cells differentiation^37^. Total lung Lin-Thy1.2^+^ cells were found downregulated in this model of asthma and not significantly regulated by NIP45 (Fig. 5e). We next analyzed Lin^−^ST2^+^ in total lung cells via flow cytometry and found that the number of these cells was increased in percentage but decreased in absolute number in asthmatic wild type mice and further significantly downregulated in asthmatic NIP45^−/−^ mice (Fig. 5f). We further investigated ILC2s in the lungs of wild-type control and Nip45^−/−^ mice. ILC2s defined as Lin^−^Thy1.2^+^KLRG1^+^ICOS^+^ cells (Fig. 5g) were significantly increased in percentage in OVA treated wild-type mice, while there was no significant induction in the absence of NIP45. When calculated as absolute number no differences among the groups were observed (Fig. S1a), similarly the gated non-ILC2 cells were induced in asthma in percentage in the lungs of naïve NIP45^−/−^ mice and not regulated when the absolute number was considered (Fig. S1b).

### Local administration of recombinant-rIL-33 reverses the protective asthmatic phenotype seen in NIP45^−/−^ mice in a murine model of allergic asthma

**Figure 6:**
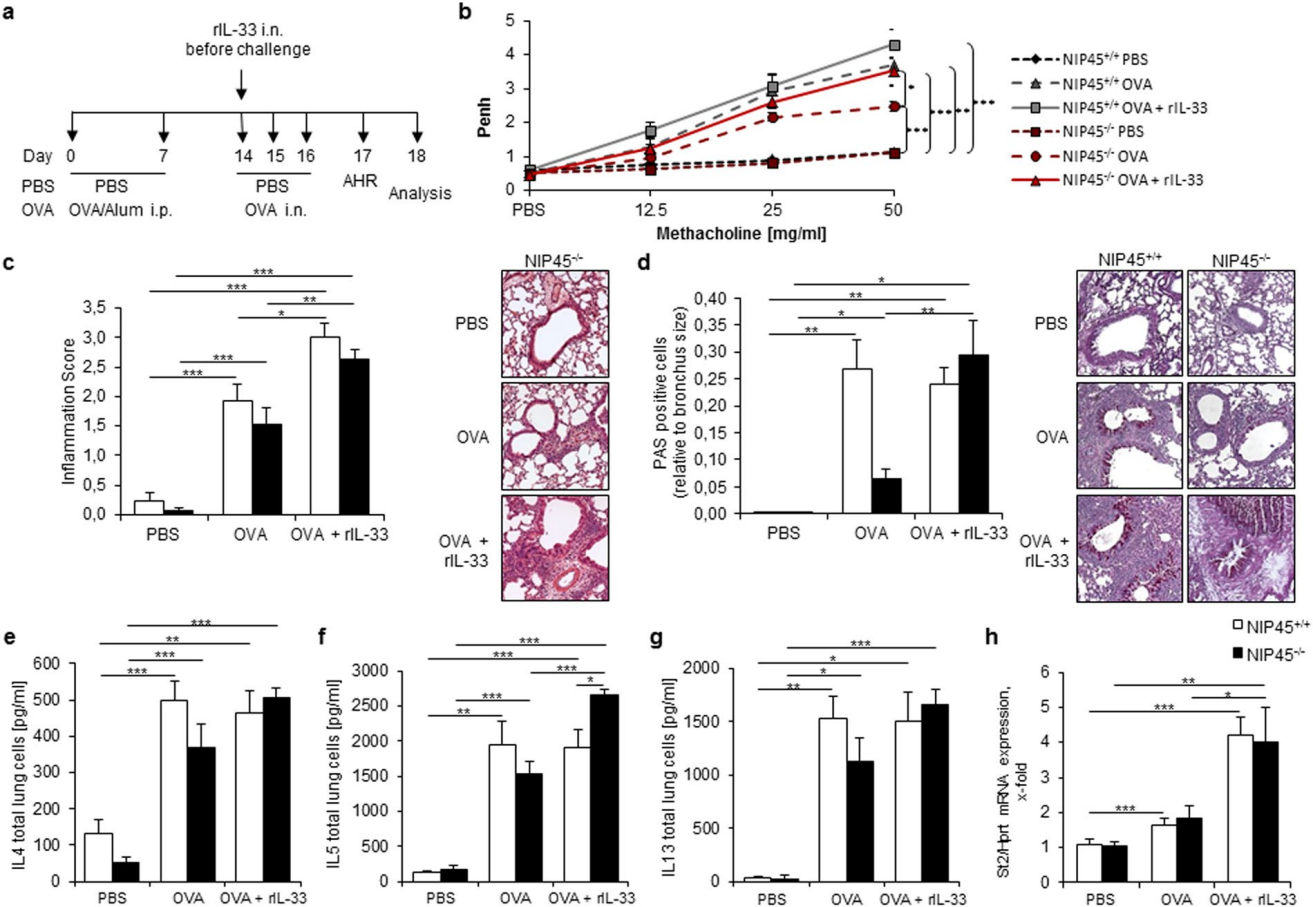
Reversed asthmatic phenotype in Nip45^−/−^ mice after local administration of recombinant IL-33 in a murine model of asthma. (**a**) Experimental design of the murine asthma model with administration of 1 µg rIL-33 on day 14, 30 minutes before the allergen challenge. (**b**) Airway resistance to increasing doses of methacholine was measured in allergen sensitized and challenged Nip45^−/−^ mice and control mice by non-invasive plethysmography. Airway resistance was analyzed as Penh. One representative experiment is shown with three to five mice per group. (**c**) Pathological score of the inflammation in the lungs of asthmatic Nip45^−/−^ mice and control mice with or without additional rIL-33 treatment and corresponding pictures of the H&E staining. (**d**) Periodic acid-Shiff (PAS) staining of lung sections from asthmatic and rIL-33 treated Nip45^−/−^ mice and control mice. (**e**–**g**) Protein levels of (**e**) IL-4, (**f**) IL-5 and (**g**) IL-13 in asthmatic Nip45^−/−^ mice treated additionally with rIL-33. (**h**) Expression of St2 mRNA in lung tissue of asthmatic Nip45^−/−^ and control mice with and without rIL-33 treatment via qPCR. n = 3 to 9 mice per group. Student’s t test was used to calculate statistical significances. *P ≤ 0.05, **P ≤ 0.01, ***P ≤ 0.001. Data are expressed as mean ± s.e.m.

To find out whether IL-33 is responsible for the decrease in Th2 cytokine production and pathological features in the lungs of asthmatic NIP45^−/−^ mice, we treated the mice once with 1 µg of recombinant r-IL-33 intranasally, 30 minutes before the first *in vivo* challenge with OVA (Fig. 6a). Next, we investigated the impact of IL-33 on airway hyperresponsiveness and found that NIP45^−/−^ mice intranasally treated with rIL-33 displayed a significant increase in the Penh value in response to methacholine compared to OVA alone, reaching Penh values comparable to the wild type asthmatic mice without exogenous rIL-33 (Fig. 6b). Next, we assessed the degree of inflammation in the lungs of the mice by H&E staining. We could observe that NIP45^−/−^ asthmatic mice that additionally received rIL-33 intranasally show a significant increase of inflammation compared to the OVA NIP45^−/−^ group (Fig. 6c). Also, the wild type asthmatic mice had a significant induction of inflammation as compared to untreated wild type asthmatic mice (Fig. 6c). The same could be observed for the PAS staining of the lung sections: asthmatic NIP45^−/−^ mice that were treated with rIL-33 had more PAS+ cells relative to bronchus size as compared to the NIP45^−/−^ mice treated with OVA alone (Fig. 6d). Subsequently, we looked for the protein levels of the Th2 cytokines IL-4, IL-5 and IL-13 in total lung cells of NIP45^−/−^ mice. We found that IL-4 was not upregulated by rIL-33, neither in wild type nor in NIP45^−/−^ asthmatic mice (Fig. 6e). In addition, IL-5 but not IL-13 was further up-regulated in asthmatic NIP45^−/−^ mice after exogenous administration of rIL-33 (Fig. 6f,g, respectively). Lastly, we investigated the mRNA expression of *St2* in the lungs of rIL-33 treated asthmatic Nip45^−/−^ mice and found a significant upregulation after rIL-33 treatment (Fig. 6h). Thus, these results demonstrate that the protective asthmatic phenotype seen in NIP45^−/−^ mice seems to be dependent on rIL-33 and ILC2s, because the local administration of rIL-33 significantly induced AHR, inflammation, mucus production, IL-5 and *St2* mRNA, the main characteristics of this disease.

### NIP45 deficient mice have decreased AHR and produce less airway mucus in a model of house dust mite (HDM)

**Figure 7:**
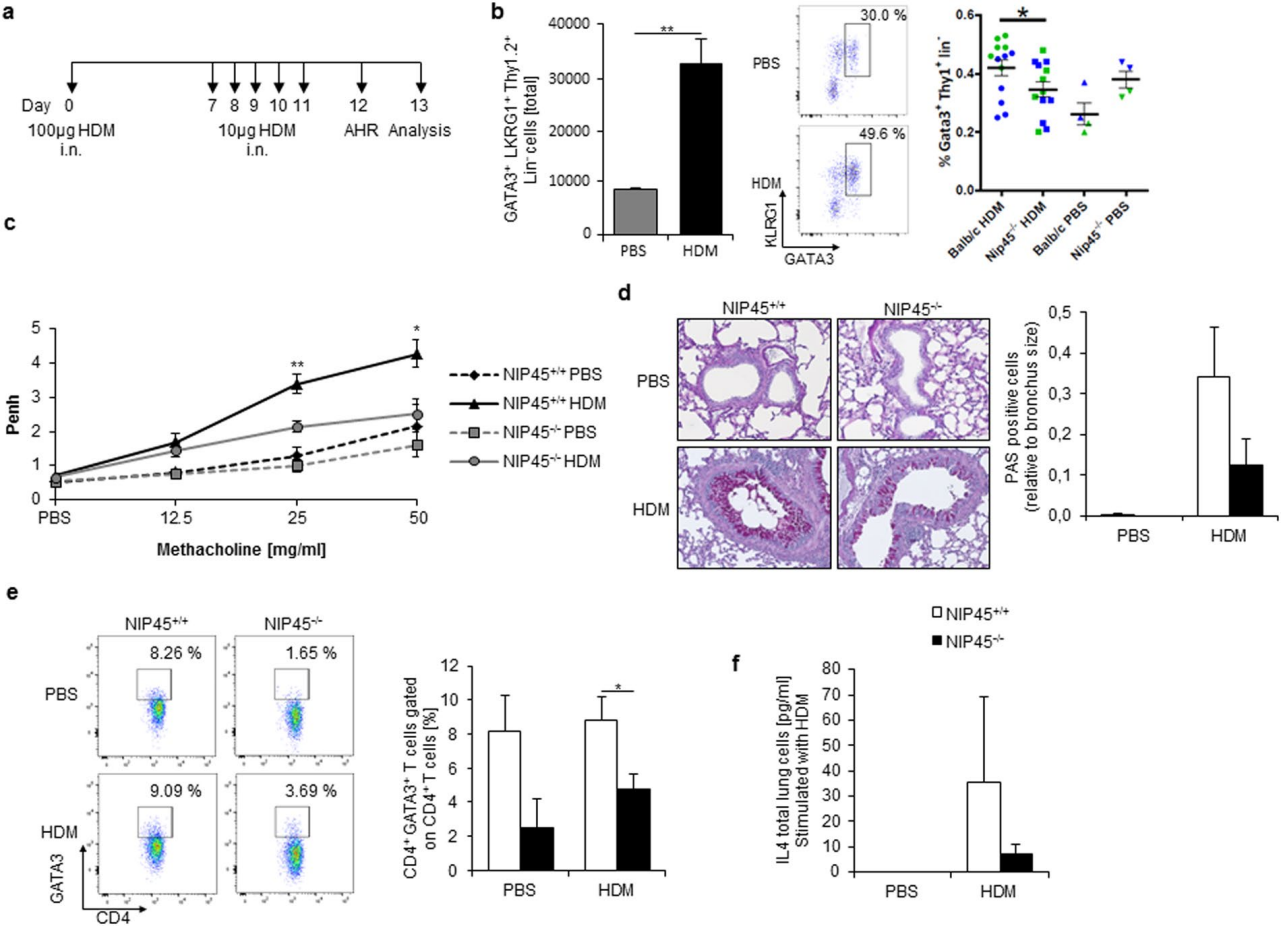
Inhibition of the asthmatic phenotype in Nip45^−/−^ mice in a HDM-induced asthma model. (**a**) Experimental design of the HDM-induced asthma model. (**b**) Flow cytometric analysis of lung GATA3+ KLRG1+ Thy1.2+ Lin- ILC2s in HDM-treated wt mice and GATA3+ Thy1.2+ Lin- ILC2s in HDM-treated wt and Nip45^−/−^ mice. n = 2 to 7 mice per group in two independent experiments (green and blue dots differentiate the groups from two separate experiments). (**c**) Airway resistance was measured as Penh with a whole-body plethysmograph to rising doses of the broncho-constrictor Methacholine. (**d**) Lung sections of untreated and HDM-treated Nip45^−/−^ and wild-type control mice were stained for Periodic acid-Shiff (PAS) to show mucus producing cells in purple. (**e**) Percentage of CD4+ GATA3+ T cells in the lungs of Nip45^−/−^ and wild-type control mice analysed via flow cytometry. (**f**) Protein concentration of IL-4 in total lung cells stimulated with HDM for 24 h measured via ELISA. Student’s t test was used to evaluate statistical significances (**c**–**f**). Mann-Whitney U test was used to calculate significances in (**b**). *P ≤ 0.05, **P ≤ 0.01, ***P ≤ 0.001. Data are expressed as mean ± s.e.m.

Because house dust mites are an important trigger of allergic asthma in humans, we next employed a more physiological model of the disease and induced asthma in the airways of both wild-type and NIP45 deficient mice by intranasal challenge with HDM extract as illustrated in Fig. 7a. Given this model does not involve systemic sensitization, it particularly involves activation of cells of innate immunity like ILC2s. Consistently, we found a significant induction of ILC2 absolute cell number in the lungs of wild type asthmatic mice (Fig. 7b). Furthermore, consistent with our previous results in the OVA induced asthma, we found a reduction in the percentage (Fig. 7b) but not in the absolute number (Fig. S1c) of ILC2s in the airways of NIP45 deficient mice. Moreover, mice deficient in NIP45 had decreased airway hyperresponsiveness (Fig. 7c) and less mucus production in the airways compared to the wild-type littermates in a model of allergic asthma (Fig. 7d). Here, GATA3, a signature transcription factor of Th2 cells and ILC2s, was downregulated in lung CD4+ T cells of HDM-treated NIP45^−/−^ mice compared to wild-type control mice (Fig. 7e). Consistent with a role of NIP45 in the activation of the *Il4* gene promoter, IL-4 was also found to be downregulated in the absence of NIP45 (Fig. 7f).

### Reduced cell number and cytokine release in ILC2s differentiated from NIP45 deficient mice after *in vivo* enrichment with a minicircle DNA IL-25-expressing vector

We next asked the reason why we obtained less ILC2 differentiation in the absence of NIP45. To investigate this point we differentiated ILC2s both in wild type and in NIP45^−/−^ mice after *in vivo* injection of a minicircle DNA vector expressing IL-25, a cytokine inducing ILC2s^38^.

**Figure 8:**
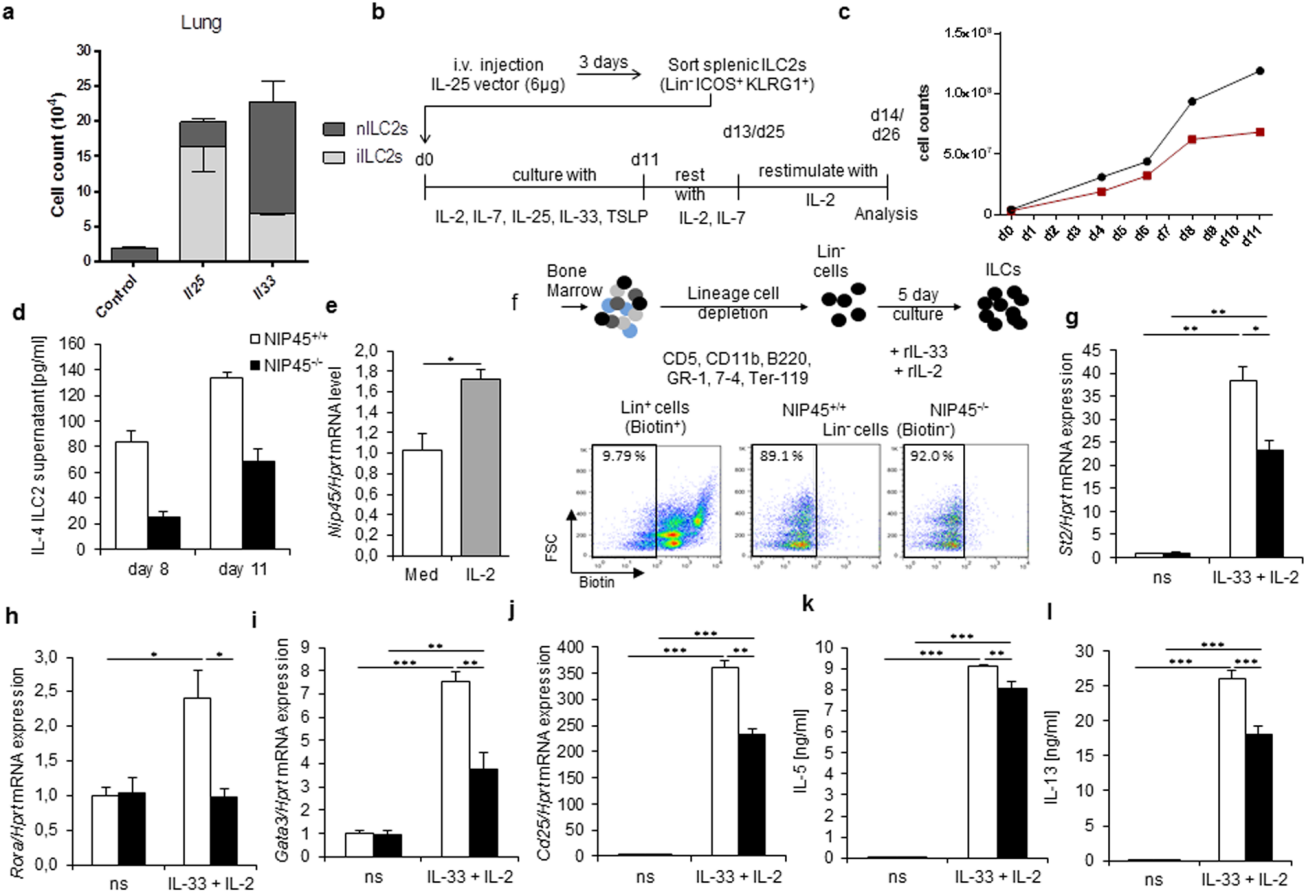
Reduced ILC2 number and specific markers and cytokine in the absence of Nip45. (**a**) Mice were hydrodynamically injected with DNA vectors encoding for Il25 or Il33 or left untreated. Lung cells were isolated after five days and nILC2s (lin^−^ CD127^+^ KLRG1^int^ ST2^+^) and iILC2s (lin^−^ CD127^+^ KLRG1^hi^ ST2^−^) analyzed. (**b**) Experimental design for ILC2 enrichment and sorting from the spleens of wt and NIP45^−/−^ mice treated *in vivo* with microcircle DNA expressing IL-25. (**c**) Cell count of wt and Nip45^−/−^ ILC2s during the expansion phase (day 0 to 11). (**d**) IL-4 protein concentration was measured in the supernatants of cultured ILC2 collected at day 8 and 11 during the expansion phase. (**e**) mRNA expression of Nip45 measured via qPCR in wt and Nip45^−/−^ ILC2s cultured for 14 days and restimulated with IL-2 for 24 h. (**f**) Experimental design of the differentiation of ILC2 from the bone marrow. Lineage negative cells were magnetically sorted the bone marrow of wt and Nip45^−/−^ mice and cultured for 5 days in medium. Afterwards RNA was extracted and an pPCR was performed. (**g**–**l**) qPCR analysis of different ILC2 markers, (**g**) St2, (**h**) RORa, (**i**) Gata3, and (**j**) Cd25. Protein levels of (**k**) IL-5 and (**l**) IL-13 were measured in the cell supernatants by ELISA. One representative experiment is shown with n = 4 mice per group. Statistical significances in this figure were evaluated with a Students t test and Mann-Whitney U test. *P ≤ 0.05, **P ≤ 0.01, ***P ≤ 0.001. Data are mean ± s.e.m.

We next analyzed mice treated with DNA vectors encoding for IL-25 and IL-33 and investigated subsets of ILC2s thoroughly. After 3 days, we sorted lung and splenic ILC2 cells Lin-ICOS+ KLRG1+ and cultured them under ILC2 differentiating conditions for 11 days (Fig. 8a,b). Here we observed that, inflammatory (i) ILC2s (lin^−^ CD127^+^ KLRG1^hi^ ST2^−^) were predominantely induced by IL-25, while natural (n) ILC2s (lin^−^ CD127^+^ KLRG1^int^ ST2^+^) preferentially increased upon IL-33 treatment in the Lung (Fig. 8a) as previously described^39^.

Then we set up a new experiment and analyzed the ILC2 after the injection of IL-25 DNA vector (Fig 8b). After 3 days we sorted lung and spleen Lin^−^ICOS^+^KLRG1^+^ILC2s and cultured them under ILC2 differentiating conditions for 11 days. During the cell growing period, we counted the spleen cells and found that at day 8, the ILC2s obtained from NIP45^−/−^ mice stopped growing (Fig. 8c). In addition, IL-4 was found significantly decreased in the absence of NIP45 in the cell supernatants of expanding ILC2s, at day 8 and day 11 (Fig. 8d). We next asked if NIP45 was expressed in ILC2s. To address this point, we measured *NIP45* mRNA levels in the ILC2 cultures derived from the wild type mice after *in vitro* ILC2s restimulation for 2 additional days with IL2 and IL-7, followed by re-stimulation for 24 hours with IL-2 (Fig. 8e). Here, we found a significant upregulation of *NIP45* mRNA as the ILC2s expanded *in vitro* (Fig. 8e). These data indicate a role of NIP45 in ILC2s. To further investigate the mechanism on how NIP45 supports the survival of ILC2s, we reasoned that NIP45 deficient ILC2s are more likely to undergo apoptosis, a form of programmed cell death. To analyze this possibility, we performed an apoptosis assay with Annexin-V and Propidium Iodide (PI) (Fig. S2a). Here we found by trend an enhanced late apoptosis in the absence of NIP45 in the culture with IL-2 (Fig. S2a).

We next investigated the proliferation of the expanded and IL-2 re-stimulated ILC2s by using Ki67 staining and found a tendential proliferative defect in ILC2s in the absence of NIP45 (Fig. S2b). Furthermore, ILC2 differentiated from NIP45 deficient mice produced less IL-13 and expressed less GATA3 as compared to those ILC2s isolated from the wild type littermates (Fig. S2c).

In conclusion, although further studies are required in this direction, NIP45 emerges as a new ILC2 marker involved in allergic asthma.

### Decreased expression of ILC2 markers in bone marrow differentiated ILCs from NIP45^−/−^ mice

As mentioned above, we demonstrated that NIP45^−/−^ mice have a reduced number of ILC2s and a decreased expression of *ST*2 after allergen sensitization and challenge. Therefore, we wanted to further analyze the differentiation of ILC2s in the absence of NIP45. Thus, we isolated bone marrow cells from wild-type and NIP45^−/−^ mice and depleted lineage positive cells. The lineage negative cells (about 90% purity as shown in Fig. 8f) were cultured for five days with medium alone or with IL-2 and IL-33 (Fig. 8f–l). We then measured some ILC2 lineage specific genes via qPCR. First, we investigated St2 mRNA expression and found that ST2 is induced after 5 days of culture with IL 33 and IL-2 in both wild-type and NIP45^−/−^ cells, but the knockout cells expressed significantly lower levels of *ST*2 mRNA (Fig. 8g). Additionally, we investigated the expression of the ILC2 signature transcription factor *RORα* and found that *Rora* mRNA is significantly increased in the stimulated wild-type cells, but not in the NIP45^−/−^ bone marrow derived ILC2 cells (Fig. 8h). Two other ILC2 markers were analyzed at RNA level, namely the transcription factor GATA3 and the IL-2R alpha chain (CD25). Both genes were significantly induced after IL-33 and IL-2 stimulation in wild-type as well as NIP45^−/−^ cells (Fig. 8i,j). NIP45^−/−^ cells expressed significantly lower levels of *GATA3* and *CD25* mRNA than wild-type cells. Since ILC2s are known to produce high amounts of the Th2 cytokines IL-13 and IL-5, we aimed to investigate their production on mRNA and protein level. We observed that both cytokines were significantly induced after stimulation with IL-33 and IL-2 in both wild-type and NIP45^−/−^ cells at protein level. Furthermore, we found that NIP45^−/−^ cells had a significantly less IL-5 and IL-13 compared to wild-type cells (Fig. 8k,l). Thus, these results further suggest that NIP45 plays an important role in the differentiation of ILC2s.

## Discussion

In the present study, we demonstrate that asthmatic pre-school children at the age of 4–6 years as well as adult asthmatics have a significantly increased expression of *NIP45* in the peripheral blood, compared to healthy children and adults, indicating that NIP45 plays an important role in the pathogenesis of asthma. In support of this hypothesis, we showed in two murine experimental models of allergic asthma that NIP45^−/−^ mice do not develop the classical characteristics of allergic asthma, such as AHR, inflammation in the lung, mucus production and local eosinophil influx. We also found that NIP45 deficiency led to a significant decrease of the Th2 cytokines IL-4, IL-5 and IL-13, which are the principle components in the onset of asthma. NIP45 usually coordinates the NFAT-driven Th2 cytokine-expression responses^3–5^. Targeted deletion of NIP45 seems to be sufficient to down-regulate the expression profile of Th2 cells in asthma. ILC2s are innate-like cells, which are known to be potent producers of the Th2 cytokines IL-5 and IL-13^40^. These mediators increase after allergen exposure and therefore play an important role in the pathogenesis of asthma^28,30,31^. They can be induced via the IL-1 family cytokine IL-33, an alarmin which is highly expressed in the lung by damaged epithelial cells. IL-33 acts through the IL-1 receptor-related protein ST2, an orphan member of the IL-1 receptor family, which is expressed on ILC2s^31,32,41^. In this study we showed that asthmatic NIP45^−/−^ mice displayed a reduced number of ILC2s due to their increased apoptosis and decreased proliferation rate. This intrinsic property of ILC2s lacking NIP45 probably has an impact on the protective phenotype observed in NIP45^−/−^ mice with reduced allergic asthma, potentially caused by the downstream NFAT-mediated reduction of Th2 cells that is dependent on the decreased number of ILC2s. To prove this hypothesis, we treated intranasally wild type and NIP45^−/−^ mice with recombinant IL-33 in our murine model of asthma. Treating the NIP45^−/−^ mice with rIL-33 reversed the protective asthmatic phenotype found in NIP45 deficiency. These mice now displayed a significant increase in AHR, lung inflammation and mucus production. Additionally, the asthmatic NIP45^−/−^ mice treated with rIL-33, had significantly elevated levels of IL-5 in the lung, indicating an effect of IL-33 on eosinophil development independent from NIP45. We also demonstrated that rIL-33 induced the transcription of the mRNA of its receptor ST2 in our model of allergic asthma, at least in the absence of NIP45. These results indicate how important IL-33 and ILC2s are in the outcome of allergic asthma, especially in the absence of NIP45. By looking further into the mechanism of ILC2 differentiation in NIP45^−/−^ and wild type mice, we intravenously injected a microcircle DNA vector expressing IL-25 *in vivo* and looked at spleen derived ILC2 differentiation. We found that NIP45^−/−^ ILC2s differentiated less and underwent apoptosis more frequently. Furthermore they proliferated less and produced less Th2 cytokines as compared to ILC2s isolated from the wild type littermates.

Thus, our data suggest that NIP45 not only plays a crucial role in the differentiation of Th2 cells but also in the differentiation of ILC2s in allergic asthma, because it is able to induce IL-4 as well as IL-13. In fact, in allergic asthma patients, inhibiting NIP45 might result in a reduction of Th2 cells and ILC2s, accompanied by a reduced asthmatic phenotype.

Although additional experiments, like adoptive transfer of ILC2 from wild type and NIP45 deficient mice KO cells should be performed in RAG KO to study asthmatic phenotype by cytokine administration like IL-33 and/or IL25, targeting NIP45 emerges as a potentially novel approach for suppression of ILC2 cells and Th2 cytokine production in asthma.

## Materials and Methods

All methods described in this manuscript were carried out in accordance with relevant guidelines and regulations. All experimental protocols were approved by a named institutional and/or licensing committee/s, including any relevant details.

### Human studies

In the human studies reported in this manuscript, informed consent was obtained from all subjects or, if subjects are under 18, from a parent and/or legal guardian.

### Cohort of pre-school children and isolation of PBMCs

In the European Study PreDicta (Post-infectious reprogramming and its association with persistence and chronicity of respiratory allergic diseases). We examined healthy and asthmatic pre-school children at the age of 4–6 years in collaboration with the children hospital in Erlangen. This cohort has been described elsewhere^42,43^. All the experiments with human samples were approved by the Ethics Committee of the Friedrich-Alexander University Erlangen-Nürnberg, Germany under the approval number ‘Re.-No. 4435 and is registered in the German Clinical Trials Register (www.germanctr.de: DRKS00004914). Furthermore, unstimulated PBMCs were isolated from the blood of the children and analyzed for gene expression.

Heparinized blood was transferred to a 15 ml sterile tube and diluted with an equal volume of PBS, inverted and carefully stratified on Ficoll-Hypaque. The peripheral blood mononuclear cells (PBMCs) were isolated and mRNA was extracted by using PeqGold RNA Pure according to the manufacturer’s protocol (PeqLab, Erlangen, Germany) or with an AllPrep DNA/RNA Mini Kit (Quiagen, Hilden, Germany). Quantitative real time PCR was performed as described below.

### Isolation of human CD4^+^ T cells from a cohort of asthmatic and control adults

Asthma Bio-Repository for Integrative Genomic Exploration (ABRIDGE) is an open-access biorepository for subjects participating in genetic studies of asthma in the EVE Consortium^44^.

Sample collection and processing of CD4^+^ T lymphocytes were carried out at each institution according to standardized and validated protocols. These samples were centralized at the Data Coordinating Center at the Channing Division of Network Medicine at Brigham and Women’s Hospital for the assembly of Asthma BRIDGE. This study was approved by local Ethic committee as previously reported. Contributing centers isolating peripheral blood CD4^+^ T lymphocytes used a modified version of the protocol previously optimized for collections in the CAMP study using Miltenyi Biotec anti-CD4+ microbeads and column separation. The modification includes isolating peripheral blood mononuclear cells (PBMCs), and then stimulating with phytohemagglutinin (PHA) prior to CD4^+^ lymphocyte isolation.

### Mice

The wild-type and Nip45^−/−^ mice (the latter being a generous gift from Prof Laurie Glimcher and Karen Mowen) had a Balb/c genetic background. Wild-type and Nip45^−/−^ mice used for the invasive body-plethysmograph had a C57BL/6 genetic background. The experiments were performed with mice aged 6–8 weeks. The animals were bred in individually ventilated cages at the animal facility adjacent to our institute and had free access to food and water. All experiments were performed with approved licenses (23-177-07/G09-1-008 from ethical review board Rheinland-Pfalz and 54-2532.1-2/10 and 54-2532.1-55/12 from the government of Mittelfranken, Bavaria).

### OVA sensitization and challenge

Wild-type mice and Nip45^−/−^ mice received an intraperitoneal injection of 100 µg OVA (Calbiochem, San Diego, CA) complexed with 10% alum (Sigma Aldrich, Steinheim, Germany) on days 0 and 7. On the days 16, 17 and 18 the animals were intranasally treated with OVA or PBS (2 mg OVA/ml PBS in solution). The airway hyperresponsiveness was measured on day 18. In a second asthma protocol mice were sensitized with OVA/Alum and challenged with OVA or sensitized with PBS/Alum and challenged with PBS. The animals were sacrificed on day 18 to isolate lung cells as described below. To measure airway reactivity, we used a non-invasive whole-body plethysmography performed with a Buxco Electronics apparatus. Airway hyperresponsiveness was analyzed as *P*_enh_. Additonally, AHR was measured invasively by using a body plethysmograph (Buxco Electronics, Inc., Wilmington, NC) as previously reported^45^.

### HDM treatment

Wild type and Nip45^−/−^ mice were treated intranasally with saline or 100 µg HDM protein obtained from *Dermatophagoides pteronyssinus* whole body extract (Greer Laboratories, Lenoir, NC) in a volume of 50 µl saline on day 0. Additionally, the mice received intranasally 10 µg HDM extract or PBS on 5 consecutive days (day 7–11). Airway hyperresponsiveness was measured 24 h after the last intranasal HDM application. On day 13, lung cells were isolated as described below.

### Collection and analysis of the BAL

Bronchoalveolar lavage was performed 24 h after the last allergen challenge, by intratracheally injecting and aspirating 0.8 ml saline twice. After its collection the BALF was centrifuged for 5 min at 1500 rpm.

The cell pellets were resuspended in 1 ml PBS and an aliquot was stained with trypan blue solution and cells were counted using a Neubauer chamber. Eosinophils and neutrophils were detected by fluorescence-activated cell sorting (FACS) analysis. The cell surface staining was performed with antibodies against CD3 (eBioscience, Frankfurt, Germany), GR-1 (BD Bioscience, Heidelberg, Germany), CD45R (eBioscience, Frankfurt, Germany) and CCR3 (BD Bioscience, Heidelberg, Germany) for 30 min at 4 °C. The samples were analyzed by using a FACS-Calibur or LSR-Fortessa (BD Bioscience, Heidelberg, Germany) and FlowJo (Treestar Inc).

### Histological analysis

Lung tissues were analyzed by using paraffin-embedded tissue slices for histology. After staining with Hematoxylin/Eosin, the pathologist performed a blind analysis of the peribronchial and perivascular inflammation, by using a semi-quantitative scoring system with a range pending between 1 (mild) and 4 (severe) as described before^33^. Additionally, to investigate mucus production, Periodic Acid-Schiff (PAS) staining was performed on paraffin-embedded lung tissue sections

### ELISA

Mouse IL-4, IFN-gamma and IL-5 were detected by using OptEIA™ sandwich ELISA kits from BD Bioscience (Heidelberg, Germany). Mouse IL-13 was detected by using a Duoset™ sandwich ELISA kit from R&D Systems (Wiesbaden, Germany).

### RNA isolation and quantitative real time–PCR

Total lung tissue was homogenized and total RNA from the tissue, total lung cells, sorted ILC2s or bone marrow cells or spleen was then extracted by using PeqGold RNA Pure according to the manufacturer’s protocol (PeqLab, Erlangen, Germany). RNA (1 µg) was reverse transcribed using the first strand cDNA synthesis kit for RT-PCR (MBI Fermentas, Sat. Leon-Rot, Germany). The resulting template-cDNA was amplified by quantitative real-time PCR using SsoFast EvaGreen Supermix (Bio-Rad Laboratories, München, Germany). The qPCR was performed with a cycle of 2 min 98 °C, 50 cycles at 5 s 95 °C, 10 s 60 °C, followed by 5 s 65 °C and 5 s 95 °C in a CFX96 Touch Real-Time PCR Detection System (Bio-Rad Laboratories, München, Germany). The primers and sequences used for mouse were as follows: *St2* (5′-GCGGAGAATGGAACCAACTA-3′, 5′-AAGCAAGCTGAACAGGCAAT-3′), *Rora* (5′-TCTCCCTGCGCTCTCCGCAC-3′, 5′-TCCACAGATCTTGCATGGA-3′), *Gata3* (5′-GTCATCCCTGAGCCACATCT-3′, 5′-TAGAAGGGGTCGGAGGAACT-3′), *Cd25* (5′-GCTCACCTGGCAACACAGATGG-3′, 5′-GGAAACAGCCGTTAGGTGAATGCT-3′), *T-bet* (5′-CCT GGA CCC AAC TGT CAA CT-3′, 5′-AAC TGT GTT CCC GAG GTG TC-3′) and *Nip45* (5′-AGGGACAAAAGCAGAAAGCA-3′, 5′-CATCCTGACAGGCAGTCTCA-3′). The mRNA of the genes of interest was normalized using the mRNA levels of the housekeeping gene *Hprt* (5′-GCCCCAAAATGGTTAAGGTT-3′, 5′-TTGCGCTCATCTTAGGCTTT-3′). For human analysis the following primers and sequences were used: *hHPRT* (5′-TGACACTGGCAAAACAATGCA-3′, 5′-GGTCCTTTTCACCAGCAAGCT-3′), *hNIP45* (5′-AGTTCTCCAGAGGCCACAGA-3′, 5′-TCAATGAGGTCCCCAGATTC-3′), *NFATc1* (5′-GCATCACAGGGAAGACCGTGTC-3′, 5′-GAAGTTCAATGTCGGAGTTTCTGAG-3′) and *T-bet* (5′-CCC TTG GTG TGG ACT GAG AT-3′, 5′-GTC GGT GTC CTC CAA CCT AA-3′.

### Flow cytometry analysis and intracellular staining

Total lung cells were stained with anti-CD4 and anti-CD8 (BD Bioscience, Heidelberg, Germany), anti-CD3, anti-CD11b, anti-CD11c, anti-CD19 and anti-FcεRI (eBioscience, Frankfurt, Germany), or anti-ST2 (MD bioscience GmbH, Egg b. Zürich, Switzerland) antibodies for 30 min at 4 °C and washed once before measuring. For intracellular staining the cells were fixed with fixation/permeabilization solution (eBioscience, Frankfurt, Germany) for 35 min at 4 °C and then stained with an antibody against GATA3 (BD Bioscience, Heidelberg, Germany) for 30 min at 4 °C in permeabilization buffer. Afterwards cells were washed once with permeabilization buffer (eBioscience, Frankfurt, Germany) and finally with PBS. For ILC2 staining, a premixed lineage cocktail (Miltenyi Biotec) containing biotinylated anti-CD3, anti-CD5, anti-CD11b, anti-CD11c, anti-B220, anti-NK1.1, anti-Ter-119, anti-Gr1, and anti-Siglec-F monoclonal antibodies was used. Streptavidin conjugated to Brilliant Violet 421 (BioLegend) was applied in a secondary staining. The following fluorochrome-tagged antibodies for surface staining were purchased from Miltenyi Biotech, unless specified otherwise: anti-Thy1.2 (30-H12), anti-KLRG1 (2F1), anti-ICOS (7E.17G9), anti-ST2 (DJ8, MD bioscience), anti-CD11b (M1/70, Invitrogen), anti-SiglecF (ES22-10D8), anti-Gr1 (1A8, BioLegend), anti-CD11c (N418, Invitrogen). The samples were analyzed by using a FACS-Calibur or LSR-Fortessa (BD Bioscience, Heidelberg, Germany) and FlowJo (Treestar Inc).

### ILC2 expansion *in vivo*, isolation and *in vitro* expansion

To expand ILC2s *in vivo*, mice were injected intravenously with 6 µg of a minicircle DNA vector expressing IL-25 as previously described^46^. Subsequently, single cell suspensions from spleen and mesenteric lymph nodes were prepared using the gentleMACS Octo device (Miltenyi Biotec) according to the manufacturer’s instructions. ILC2s were identified using the following sort panel: ICOS VioBlue^+^ (7E.17G9, Miltenyi Biotec), KLRG1 PE^+^ (REA1016, Miltenyi Biotec), CD5 FITC^−^ (REA421, Miltenyi Biotec), CD45R FITC^−^ (REA755, Miltenyi Biotec), NKp46 FITC^−^ (REA815, Miltenyi Biotec), CD49b PE-Vio770^−^ (REA981, Miltenyi Biotec), CD11b APC-Vio770^−^ (REA592, Miltenyi Biotec), CD11c APC-Vio770^−^ (N418, Miltenyi Biotec), CD3 FITC^−^ (17A2, BioLegend) and FcεR1a PE-Cy7^−^ (MAR-1, Invitrogen). FACS purification was achieved by using a MoFlo Astrios EQ device (Beckman Coulter) at the Core Unit Cell Sorting Erlangen. Subsequently, ILC2s were expanded *in vitro* for 11 days by cell culture in DMEM GlutaMAX medium supplemented with 10% FBS, 1x MEM NEA (Gibco), 1 mM sodium pyruvate (Gibco), 20 mM Hepes (Carl Roth), 50 µM 2-Mercaptoethanol (Sigma-Aldrich), 1% Penicillin-Streptomycin (Sigma-Aldrich) and recombinant IL-7, IL-25, IL-33 (50 ng/ml each, Immunotools), IL-2 (50 ng/ml, BioLegend) and TSLP (20 ng/ml, Invitrogen). Thereafter, ILC2s were restimulated in cell culture medium supplemented with IL-2 and IL-7 (10 ng/ml) for at least 48 h before re-stimulation with IL-2 for 24 hours. For flow cytometric analyses, the following fluorochrome-tagged antibodies were used for cell surface staining: anti-CD4 (RM4-5, BD Biosciences) and anti-CD45 (30F11, Miltenyi). For intracellular cytokine stainings, cells were stimulated for 4 hours with the Cell Stimulation Cocktail plus protein transport inhibitors (Invitrogen) prior to surface stainings. Depending on the experiment, cells were subsequently fixed and permeabilized using the Foxp3 Transcription Factor Staining Buffer Kit (Invitrogen) according to manufacturer’s instructions followed by intracellular staining with fluorochrome-coupled anti-GATA3 (REA174, Miltenyi Biotec), anti-IL-13 (eBio13A, eBioscience) or anti-Ki-67 (16A8, BioLegend) antibodies. Apoptosis was investigated using the Annexin V Kit with propidium Iodide (PI; Miltenyi Biotec) according to manufacturer’s instructions. The samples were analyzed by using a FACS Canto II cell analyzer (BD Bioscience, Heidelberg, Germany) and FlowJo (Treestar Inc).

### ILC2 differentiation from the bone marrow

Hind legs of the mice were prepared and bone marrow cells were flushed from the tibias and femurs with sterile PBS. Cells were then centrifuged for 5 min at 4 °C and 1500 rpm and resuspended in RPMI 1640 culture medium supplemented with 10% FBS, 1% penicillin/streptomycin, 1% L-Glutamin and 50 µmol β-mercaptoethanol. Afterwards, lineage positive cells were depleted with a Lineage cell depletion kit from Miltenyi Biotec (Bergisch Gladbach, Germany) according to the manufacturer’s instructions. Purity of the cells was measured by flow cytometry with an anti-Biotin antibody conjugated with APC and 20 min incubation. Then, cells were cultured for 5 days at a density of 1 × 10^6^ cells/2 ml medium alone or medium with 10 ng/ml recombinant IL-33 (ImmunoTools, Friesoythe, Germany) and 10 ng/ml recombinant IL-2 (ImmunoTools, Friesoythe, Germany). Supernatants were collected and analysed by ELISA and RNA was extracted from cell pellets as mentioned above.

### Statistical analysis

Statistical differences were evaluated for significance (P < 0.05) by the Student’s two-tailed t test for parametric data. Additionally, we used the Mann-Whitney U test to calculate statistical significances. Data are given as mean values ± s.e.m.

Gene expression analysis in the subjects from Asthma BRIDGE biorepository was done in R using Bioconductor limma package. A linear model was fitted to the expression values for each probe to assess the significance of differential expression between adult asthmatics and healthy controls with the inclusion of potential confounders. An Empirical Bayes method using eBays function was used to obtain moderated t-statistics for *NIP45*^47^.

## Supplementary information

Supplementary data
