# Supplementary material for: Targeted deletion of NFAT-Interacting-Protein-(NIP) 45 resolves experimental asthma by inhibiting Innate Lymphoid Cells group 2 (ILC2)
Source: Sci Rep. 2019 Oct 30;9:15695. doi: 10.1038/s41598-019-51690-z (PMC6821848; doi:10.1038/s41598-019-51690-z)
Supplement: Supplementary file 1 — Supplementary data [file 41598_2019_51690_MOESM1_ESM.pdf]

Targeted deletion of NFAT-Interacting-Protein-(NIP) 45 resolves experimental asthma by inhibiting Innate Lymphoid Cells group 2 (ILC2)

Sonja Koch, PhD[1], Lisa Knipfer[2], Julia Kölle[1], Hooman Mirzakhani, MD[3], Anna Graser, PhD[1], Theodor Zimmermann, MD[4], Alexander Kiefer, MD[4], Volker O. Melichar, MD [4], Wolfgang Rascher, MD[4], Nikolaos G.Papadopoulos MD [5,6], Ralf J. Rieker, MD[7], Benjamin A. Raby, MD MPH [3], Scott T. Weiss, MD MS [3], Stefan Wirtz, PhD[2], and Susetta Finotto, PhD[1]*.

[1] Department of Molecular Pneumology, Friedrich-Alexander University Erlangen-Nürnberg, Erlangen, Germany

[2] Department of Medicine 1 - Gastroenterology, Pneumology and Endocrinology, Friedrich-Alexander-University Erlangen-Nürnberg, Erlangen, Germany

[3] Channing Division of Network Medicine, Brigham and Women's Hospital, Harvard Medical School, Boston, MA.

[4] Department of Pediatrics and Adolescent Medicine, Universitätsklinikum Erlangen, Erlangen, Germany.

[5]Allergy and Clinical Immunology Unit, 2nd Pediatric Clinic, National and Kapodistrian University of Athens, 11527 Athens, Greece.

[6]Division of Infection, Immunity & Respiratory Medicine, University of Manchester, UK.

[7]Institute of Pathology, Friedrich-Alexander-Universität Erlangen-Nürnberg (FAU), Erlangen, Germany

Running Title: NIP45 regulates allergic asthma

*Corresponding Author
Prof. Susetta Finotto, PhD
Laboratory of Cellular and Molecular Lung Immunology
Department of Molecular Pneumology,
Universitätsklinikum Erlangen, Hartmannstraße 14
91052 Erlangen, Germany
Phone: 0049-9131-8542454
Fax: 0049-9131-8535981
Email: susetta.finotto@uk-erlangen.de

Table S1: Clinical data of the analyzed children with the PreDicta study. Clinical data of healthy and asthmatic children at the age of 4-6 years that took part in the European study PreDicta; n.d. = not done

Supplementary Figure legends

Group / Patient	Age [years]	Gender	Skin Prick Test	Atopic Eczema	Treatment	*NIP45* mRNA expression	*NFATc1* mRNA expression
**Healthy**							
1	5	female	Negative	no	-	0,145	0,350
2	4	male	n.d.	no	-	0,278	0,190
3	6	male	n.d.	yes	-	1,043	0,950
4	4	female	n.d.	no	-	0,638	0,700
5	6	male	n.d.	no	-	0,522	0,330
6	4	male	negative	no	-	0,927	0,670
7	5	female	n.d.	no	-	2,772	0,920
8	5	female	alternaria	no	-	1,415	0,660
9	4	female	negative	no	-	1,742	1,640
10	5	male	negative	no	-	2,130	1,030
11	4	male	negative	no	-	5,318	2,260
**Mean**	**4,7**					**1,539**	**0,882**
**SEM**	**0,24**					**0,449**	**0,183**
**Asthma**							
1	6	male	alternaria, cat, grass	yes	steroid	47,535	80,220
2	6	male	alternaria, birch, grass	yes	steroid	20,980	51,830
3	5	female	cat	no	steroid	41,669	40,670
4	6	male	alternaria, ambrosia, cat, grass, house dust mite	yes	steroid	34,557	34,440
5	5	male	cat	no	steroid	13,746	13,510
6	5	female	alternaria	no	steroid	32,693	20,330
7	5	male	grass	yes	steroid	14,132	24,860
8	6	female	birch, grass	yes	non-steroid	23,603	36,400
9	6	female	birch, cat, grass, house dust mite	yes	steroid	31,579	27,020
10	4	female	negativ	no	steroid	0,467	0,710
11	4	male	n.d.	no	steroid	0,342	0,460
12	5	male	cat, grass, house dust mite	no	non-steroid	0,304	0,260
13	5	male	alternaria, birch, cat, grass, house dust mite	yes	non-steroid	0,758	1,090
14	5	male	alternaria, ambrosia, birch, cat, grass, house dust mite	yes	non-steroid	1,311	0,580
15	4	male	birch	no	steroid	1,058	0,720
16	5	female	n.d.	no	non-steroid	4,441	2,270
17	5	female	negative	no	steroid	2,058	0,780
**Mean**	**5,1**					**15,955**	**19,774**
**SEM**	**0,17**					**3,996**	**5,620**

Supplementary Figure legends

FIG S1. Analysis of ILC2 numbers and non-ILC2 in the lungs of allergen sensitized and challenged Nip45$^{-/-}$ mice.**(a, b)** Flow cytometry analysis of Lin- Thy1.2+ KLRG1+ ICOS+ ILC2s (a) and Non-ILC2s (Lin-Thy1.2- cells and Lin+ cells) **(b)** in total lung cells isolated from wild-type and Nip45-/- mice after induction of asthma. n = 3-5 mice per group. **(c)** Cells were pre-gated on the lymphocyte population and lineage- Thy1+ and lineage+ Thy1+ cells analyzed for Gata3 and CD4 expression.

d) Absolute ILC2 number of the experiments shown in Fig 5b. Statistical significances in this figure were evaluated with a Students t test. * $P \le 0.05$, ** $P \le 0.01$, *** $P \le 0.001$. Data are mean ± s.e.m.

Fig S2. **(a)** Flow cytometric analysis of AnnexinV+ PI+ ILC2s performed 24h (day 26) after restimulation with IL-2. **(b)** Proliferation was analysed with Ki67+ ILC2s via flow cytometry 24h after restimulation with IL-2. **(c)** GATA3+ and IL-13+GATA3+ ILC2s were analysed 24h after restimulation via flow cytometry.

Figure S1

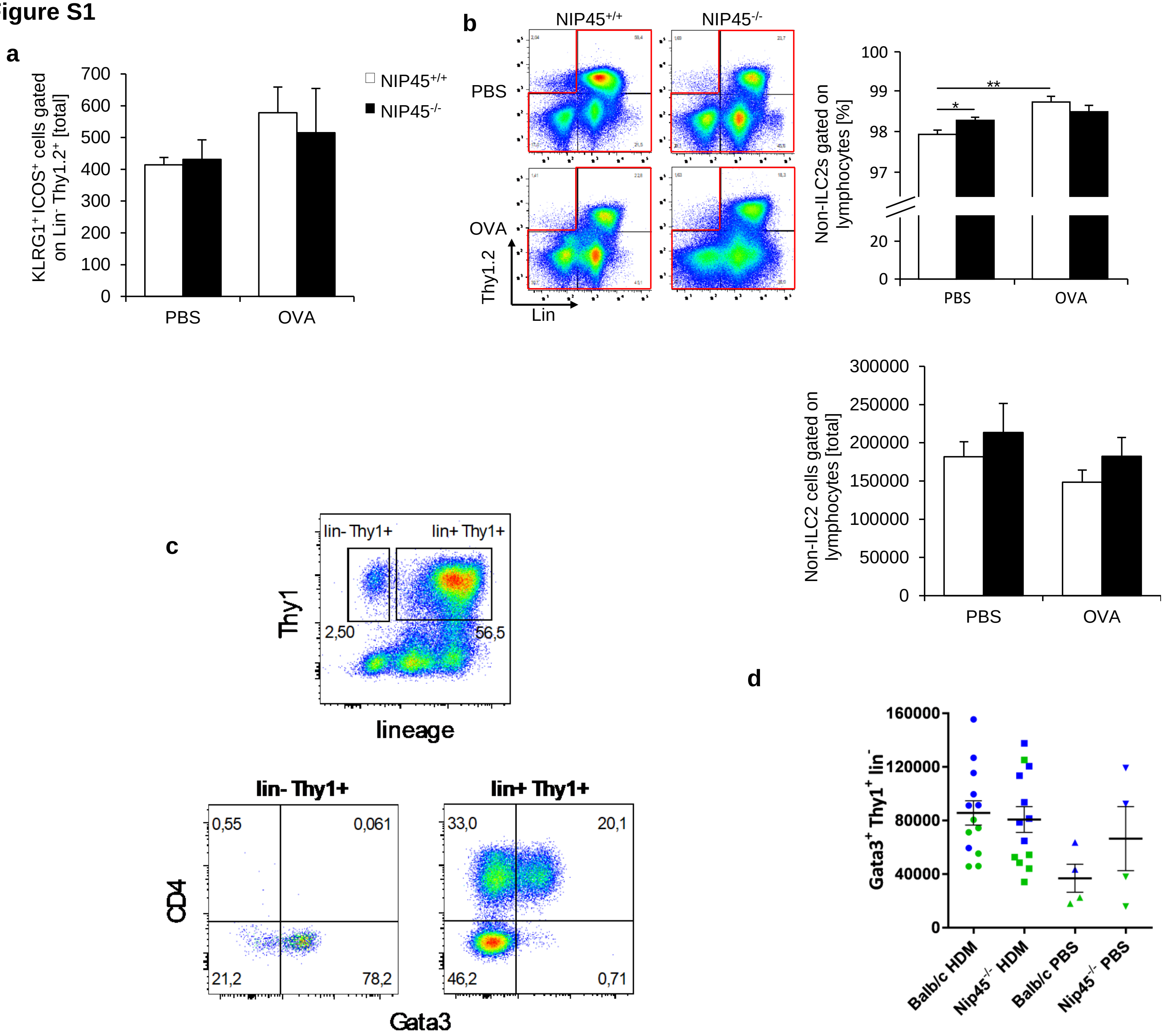

a
KLRG1+ ICOS+ cells gated on Lin- Thy1.2+ [total]
700
600
500
400
300
200
100
0
PBS
OVA
NIP45+/+
NIP45-/-
b
NIP45+/+
NIP45-/-
PBS
OVA
Thy1.2
Lin
Non-ILC2s gated on lymphocytes [%]
100
99
98
97
20
0
*
**
PBS
OVA
Non-ILC2 cells gated on lymphocytes [total]
300000
250000
200000
150000
100000
50000
0
PBS
OVA
c
lin- Thy1+
lin+ Thy1+
Thy1
2,50
56,5
lineage
lin- Thy1+
lin+ Thy1+
CD4
0,55
0,061
21,2
78,2
33,0
20,1
46,2
0,71
Gata3
d
Gata3+ Thy1+ lin-
160000
120000
80000
40000
0
Balb/c HDM
Nip45-/- HDM
Balb/c PBS
Nip45-/- PBS

Figure S2

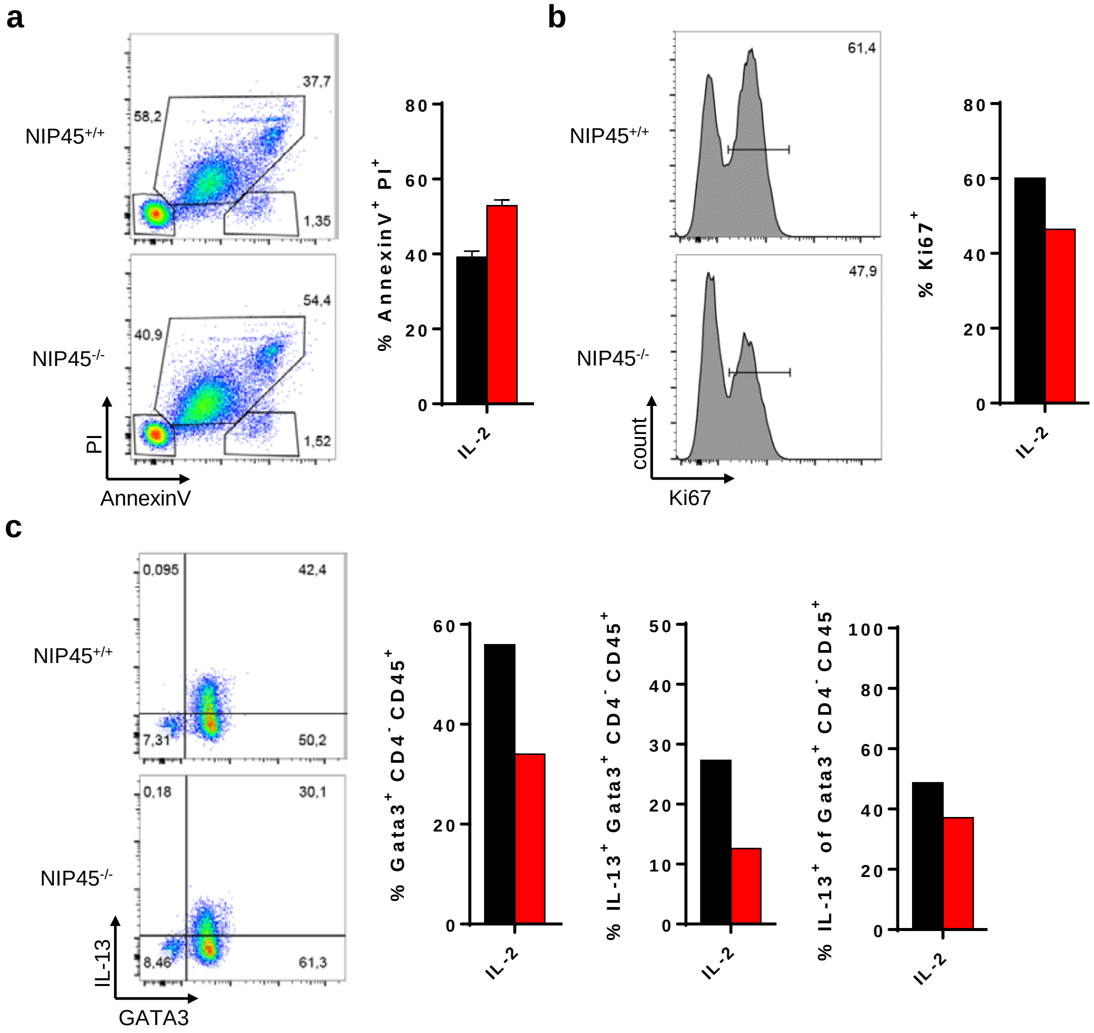

a
NIP45+/+
NIP45-/-
58,2
37,7
1,35
40,9
54,4
1,52
PI
AnnexinV
% AnnexinV+ PI+
IL-2
b
NIP45+/+
NIP45-/-
61,4
47,9
count
Ki67
% Ki67+
IL-2
c
NIP45+/+
NIP45-/-
0,095
42,4
7,31
50,2
0,18
30,1
8,46
61,3
IL-13
GATA3
% Gata3+ CD4- CD45+
% IL-13+ Gata3+ CD4- CD45+
% IL-13+ of Gata3+ CD4- CD45+
IL-2
